# Pregnant Mothers with Resolved Anxiety Disorders and Their Offspring Have Reduced Heart Rate Variability: Implications for the Health of Children

**DOI:** 10.1371/journal.pone.0083186

**Published:** 2013-12-10

**Authors:** Marijke A. K. A. Braeken, Andrew H. Kemp, Tim Outhred, Renée A. Otte, Geert J. Y. J. Monsieur, Alexander Jones, Bea R. H. Van den Bergh

**Affiliations:** 1 Department of Developmental Psychology, Tilburg University, Tilburg, The Netherlands; 2 REVAL Rehabilitation Research Center, Biomedical Research Institute, Faculty of Medicine and Life Sciences, Hasselt University, Hasselt, Belgium; 3 SCAN Research & Teaching Unit, School of Psychology, University of Sydney, Sydney, New South Wales, Australia; 4 CADE Clinic, Discipline of Psychiatry, Sydney Medical School, University of Sydney, Sydney, New South Wales, Australia; 5 Hospital Universitário, University of São Paulo, São Paulo, Brazil; 6 ERISS, European Research Institute in Service Science, Department of Information Management, Tilburg University, Tilburg, The Netherlands; 7 LIRIS, Department of Information Management, KU Leuven, Leuven, Belgium; 8 Institute of Cardiovascular Science, University College London, London, United Kingdom; 9 Department of Psychology, KU Leuven, Leuven, Belgium; University of Pennsylvania, United States of America

## Abstract

**Objective:**

Active anxiety disorders have lasting detrimental effects on pregnant mothers and their offspring but it is unknown if historical, non-active, maternal anxiety disorders have similar effects. Anxiety-related conditions, such as reduced autonomic cardiac control, indicated by reduced heart rate variability (HRV) could persist despite disorder resolution, with long-term health implications for mothers and children. The objective in this study is to test the hypotheses that pregnant mothers with a history of, but not current anxiety and their children have low HRV, predicting anxiety-like offspring temperaments.

**Methods:**

The participants in this case-control study consist of 56 women during their first trimester and their offspring (15 male, 29 female). Women had a history of an anxiety disorder (n=22) or no psychopathology (n=34) determined using the Mini-International Neuropsychiatric Interview. The main outcome measures were indices of autonomic cardiac control including root mean square of successive differences (RMSSD) and high frequency (HF) variability. Children’s fearfulness was also assessed using the Laboratory Temperament Assessment Battery (Lab-TAB)-Locomotor Version.

**Results:**

HRV was lower in women and children in the past anxiety group compared to controls. HRV measures for mothers and children were positively correlated in the anxiety group only. In all children, low HRV measures at 2-4 months were associated with a higher chance of fearful behavior at 9-10 months.

**Conclusions:**

Pregnant women with previous but not current anxiety and their children have low HRV. Children with low HRV tend to show more fearfulness. These findings have implications for identifying children at risk of anxiety disorders and point to possible underlying mechanisms of child psychopathology.

## Introduction

More than one third of adults have a history of anxiety disorders and women are twice as likely to experience such disorders [1]. Though the presence of anxiety disorders during pregnancy has an impact on both mothers and their offspring [2,3], it is not known if a resolved anxiety disorder is similarly important. Mood and anxiety disorders are associated with abnormal autonomic nervous system (ANS) function, indexed by reduced heart rate variability (HRV). Reduced HRV is a marker of physical and mental ill health and has been identified as a risk factor for various diseases, including cardiovascular disease (CVD) and mortality [4-8]. If autonomic abnormalities (e.g., low HRV) also persist in pregnant women who have had an anxiety disorder, these could have important, under-appreciated health risks in both, mothers and their children. However, it is not known whether past mental disorders could affect pregnant women and their children via reduced HRV, or other uncharacterized mechanisms. HRV of the developing fetus is altered in the offspring of mothers with a number of psychiatric conditions, including anxiety disorders, and these differences persist postnatally [9-11]. This suggests that the development of the ANS may be susceptible to the influence of maternal characteristics, with potential long-term consequences for the health of the offspring. Therefore, improved understanding of the relationship between maternal psychiatric illness, HRV and infant physical and mental wellbeing is an important goal. 

In this study, we aimed to assess the physiological correlates of past maternal anxiety disorders in both pregnant mothers and their offspring, focusing on HRV as a potential shared risk factor for ill health and abnormal autonomic function. To optimize our study, HRV was measured at rest and during mental stress. Although associations with HRV are apparent at rest [12,13], they are often more evident when participants are exposed to stress [14], and anxious individuals may be particularly sensitive to such stress exposure [15,16]. We assessed HRV in mothers with a remitted anxiety disorder and healthy controls in their first trimester of pregnancy, a critical period of fetal development. We then assessed the HRV of their offspring at 2-4 months of age and the child’s temperament at 9-10 months. 

We hypothesized the following: 1) mothers with a past, but no current, anxiety disorder will demonstrate abnormal autonomic physiology (particularly low HRV); 2) these differences will be more apparent under stress than under rest; 3) maternal HRV will be positively correlated with offspring HRV; 4) offspring of mothers with a history of an anxiety disorder will display abnormal autonomic physiology (low HRV) and; 5) this will be associated with temperamental disturbances in the offspring that might be considered predictive of later psychopathology.

## Materials and Methods

### Participants

**Figure 1:**
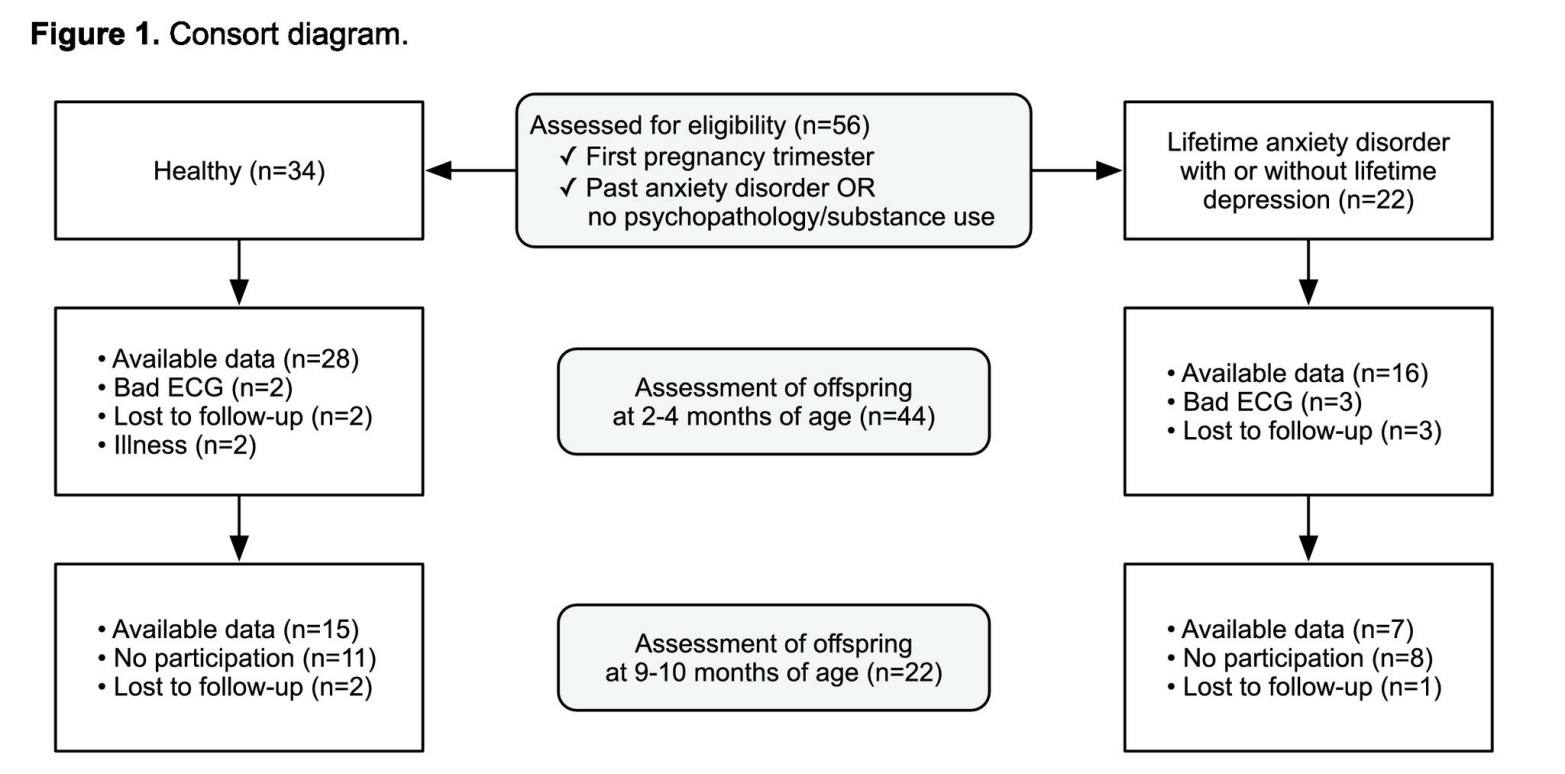
Consort diagram. This figure shows the loss to follow-up or attrition in the study participants.

Participants in the present study were recruited as part of the Prenatal Early Life Stress (PELS) study by midwives and hospitals, between May 2009 and July 2010. The overarching goal of PELS is to study the associations between prenatal risk factors, birth outcome and altered child psychophysiology and neurodevelopment. For the purposes of the present study, a group of women with a past, but no current, anxiety disorder (PAD group) and a healthy control group without any history of psychopathology were defined. No participants with a current diagnosis of an anxiety disorder were available from the PELS study. PAD included panic disorder, agoraphobia, generalized anxiety disorder, social phobia, obsessive-compulsive disorder and posttraumatic stress disorder. In the PAD group, 17 women had only one of these anxiety diagnoses, while five women had two of them. Nine women also had a history of comorbid depression and five had a history of a comorbid eating disorder. None of the participants were under treatment for a current mental disorder, used cardiovascular medications or antidepressants. Loss to follow-up or attrition due to data loss in the groups is shown in Figure 1.

### Ethics Statement

The Medical Ethics Committee of the St. Elisabeth hospital in Tilburg (the Netherlands) approved the study. All participants provided informed, written consent for themselves and their children.

### Procedures

#### Psychological and Psychiatric Assessment of Mothers

The Mini-International Neuropsychiatric Interview 6.0 [17] was administered within the second pregnancy trimester to assess past maternal psychopathology. This is a structured interview based on Diagnostic and Statistical Manual of Mental Disorders, 4th Edition (DSM-IV) criteria. In the first pregnancy trimester women completed the state anxiety subscale of the State Trait Anxiety Inventory [18]. The women rated themselves on twenty items on a four-point Likert scale ranging from “(1) - not at all” to “(4) - very much”, resulting in an anxiety total score ranging between 20 and 80. 

#### Relaxation and Stress Tasks

In the first pregnancy trimester each mother undertook a 25-minute task that consisted of five testing phases, lasting 5 minutes each. Stress was induced in the second and fourth phase, with the remainder being relaxation phases. During the stress phases, participants were asked to solve complex mental arithmetic problems, involving five mathematical operations on 2-3 digit numbers without verbalization (e.g., (361+11)÷(3× 4)+137). They were asked to choose the correct answer from three possibilities presented by a computer. Feedback on the task was given after completion of the last phase [19]. Participants viewed peaceful pictures and listened to restful music during the relaxation phase [20].

#### ECG Recording

During the relaxation and stress tasks maternal ECG was recorded using the Vrije Universiteit Ambulatory Monitoring system (VU-AMS) [21] in a three Ag/AgCl electrode placement configuration: (1) on the sternum over the ﬁrst rib between the two collarbones, (2) at the apex of the heart over the ninth rib on the left lateral margin of the chest, and (3) at the lower right abdomen (ground electrode). The skin was cleaned with alcohol to ensure impedance was low. Five-minute HRV measurements were determined for the relaxation and stress phases separately.

Children’s ECGs were collected when they were 2-4 months of age. The active electrode was placed on the child’s thorax, while the reference and ground electrode were on an EEG cap [22]. These recordings were made using a Biosemi ActiveTwo biopotential measurement system. Although some data were discarded due to motion artifacts, HRV measurements were determined from the average of 3 to 5 blocks of ECG, lasting 2.5 minutes each. 

#### Behavioral Assessment of the Children

An additional assessment of child behavior at 9-10 months of age was conducted using the Laboratory Temperament Assessment Battery (Lab-TAB)-Locomotor Version [23], a more ecologically valid and less biased assessment of child behavior and temperament than parental-report measures with demonstrable clinical value [24]. The child was presented with a robotic dog (the unpredictable mechanical toy paradigm from the fear subscale) that was barking and walking towards them. Based on the children’s facial expression, body posture, vocalizations, and escape behavior, indicators of fear were assessed for the first 20 seconds [25]. A researcher, blind to maternal data, rated the recorded videotapes using the standard scoring procedures. Observed fearfulness was calculated from the fear indicators. As the fearfulness measure could not be normalized, children with a score of 0 or 1 were categorized as not fearful, while children with a score above 1 were considered fearful.

### Data Processing and Analyses

ECG data were processed using custom software written in Matlab (Mathworks, Natick, USA) to obtain indices of parasympathetic ANS activity, according to published standards [26]. These measures were root mean square of successive differences (RMSSD) and high frequency (HF) variability. ECG beat detection was carried out with the Hilbert transform algorithm [27]. Potentially erroneous beat detections were identified using a standard approach and screened for validity [28]. As HRV measures were right-skewed, they were natural log transformed to normality. Data for three mothers were excluded due to extreme values; visual inspection of the ECGs suggested that these outliers were due to cardiac arrhythmias.

Statistical analyses were performed using SPSS 19 [29]. A repeated measures ANOVA was used to examine the differences between mothers’ HRV during the various phases of the mental arithmetic task across the two groups (healthy group versus PAD group). Mother’s age and pre-pregnancy BMI were included as covariates. To account for mothers’ state anxiety, we conducted a propensity score matching to balance state anxiety, age and BMI in both groups. This procedure was executed using a custom designed plugin [30] for IBM SPSS Statistics. Logistic regression is used to determine a predicted score that relates to the propensity for participants to belong to a particular group based on a given set covariates. The matching algorithm was fine-tuned by discarding units outside the area of common support to improve balance on covariates, and using a ‘caliper’ of 0.5 – this is the standard deviation of the logit of the propensity score – to prevent ‘bad’ matches. After the propensity score is estimated, cases with the closest score are matched using a simple 1:1 nearest neighbor matching routine based on a ‘greedy’ matching algorithm. A series of model adequacy checks were then performed including inspection of numerical balance measures, diagnostic plots and re-examination of group differences across the covariates entered into the PS analysis. Next, mothers’ state anxiety was added as covariate in the repeated measures ANOVA. Independent *t*-tests were conducted to investigate group differences in children’s HRV. Linear regression analyses were conducted to determine whether maternal HRV was associated with that of their children, and logistic regression analyses to determine whether children’s HRV at 2-4 months of age was associated with fearfulness at 9-10 months of age. Regression analyses were controlled for sex, age, birth weight, and gestation length of the child and mother’s state anxiety. The effect of offspring HRV on the continuous variable fearfulness was also tested for significance by generating bootstrapped 95% confidence intervals as the fearfulness variable was not normal.

## Results

### Participant Characteristics

Characteristics of mothers and children in the healthy and PAD groups are shown in Table 1. Importantly, no differences between groups were observed for variables that might have a confounding influence on HRV, except for mothers’ state anxiety. Age and BMI of mothers, and age, sex distribution, birth weight, fearfulness and gestation length of the children did not differ significantly between the groups. Propensity score matching identified and matched 18 mothers in each group, providing a matched sample across all of the critical confounding factors, including mothers’ state anxiety. 

**Table 1:** Comparison of mean (SD) characteristics of mothers and children in the healthy group versus the past anxiety disorder group.

	**Healthy**	**Past anxiety**	***p*-value**
***Mothers***			
N	34	22	
AGE (yr)	34.31 (3.14)	33.47 (4.95)	.44
BMI (kg/m^2^)	24.51 (4.98)	24.13 (3.13)	.75
STAI (state)^a^	33.29 (5.79)	39.05 (6.98)	.001
RMSSD Phase 1 (rest) (ln ms)	3.46 (0.53)	3.2 (0.44)	.06
RMSSD Phase 2 (stress) (ln ms)	3.31 (0.48)	2.92 (0.48)	.005
RMSSD Phase 3 (rest) (ln ms)	3.38 (0.45)	3.15 (0.51)	.08
RMSSD Phase 4 (stress) (ln ms)	3.28 (0.43)	3.08 (0.51)	.13
RMSSD Phase 5 (rest) (ln ms)	3.47 (0.46)	3.17 (0.45)	.02
HF Phase 1 (rest) (ln ms^2^)	6.16 (1.02)	5.69 (0.77)	.07
HF Phase 2 (stress) (ln ms^2^)	5.92 (0.92)	5.25 (0.86)	.009
HF Phase 3 (rest) (ln ms^2^)	5.99 (0.91)	5.49 (0.93)	.05
HF Phase 4 (stress) (ln ms^2^)	5.97 (0.72)	5.39 (0.8)	.006
HF Phase 5 (rest) (ln ms^2^)	6.25 (0.84)	5.54 (0.85)	.003
***Children at 2-4 months***			
N	28	16	
Age (wk)	15.2 (5.1)	16.6 (6.2)	.41
Sex	8 boys, 20 girls	7 boys, 9 girls	.31^f^
Gestation (wk)	39.4 (1.1)	39.1 (2.1)	.54
Birth weight (g)	3330 (348)	3269 (562)	.87
RMSSD Rest (ln ms)	2.47 (0.51)	2.16 (0.44)	.048
HF Rest (ln ms^2^)	4.43 (1.04)	3.78 (1.00)	.048
***Children at 9-10 months***			
N	15	7	
Age (wk)	35.7 (3.1)	35.7 (3.6)	>.99
Sex	4 boys, 11 girls	4 boys, 3 girls	.17^b^

^a^ Analysis focused on the state anxiety subscale of the STAI (20 items measuring the intensity of anxiety-related symptoms).

^b^ X^2^ test

### Impact of Past Anxiety Disorder in Women

**Figure 2:**
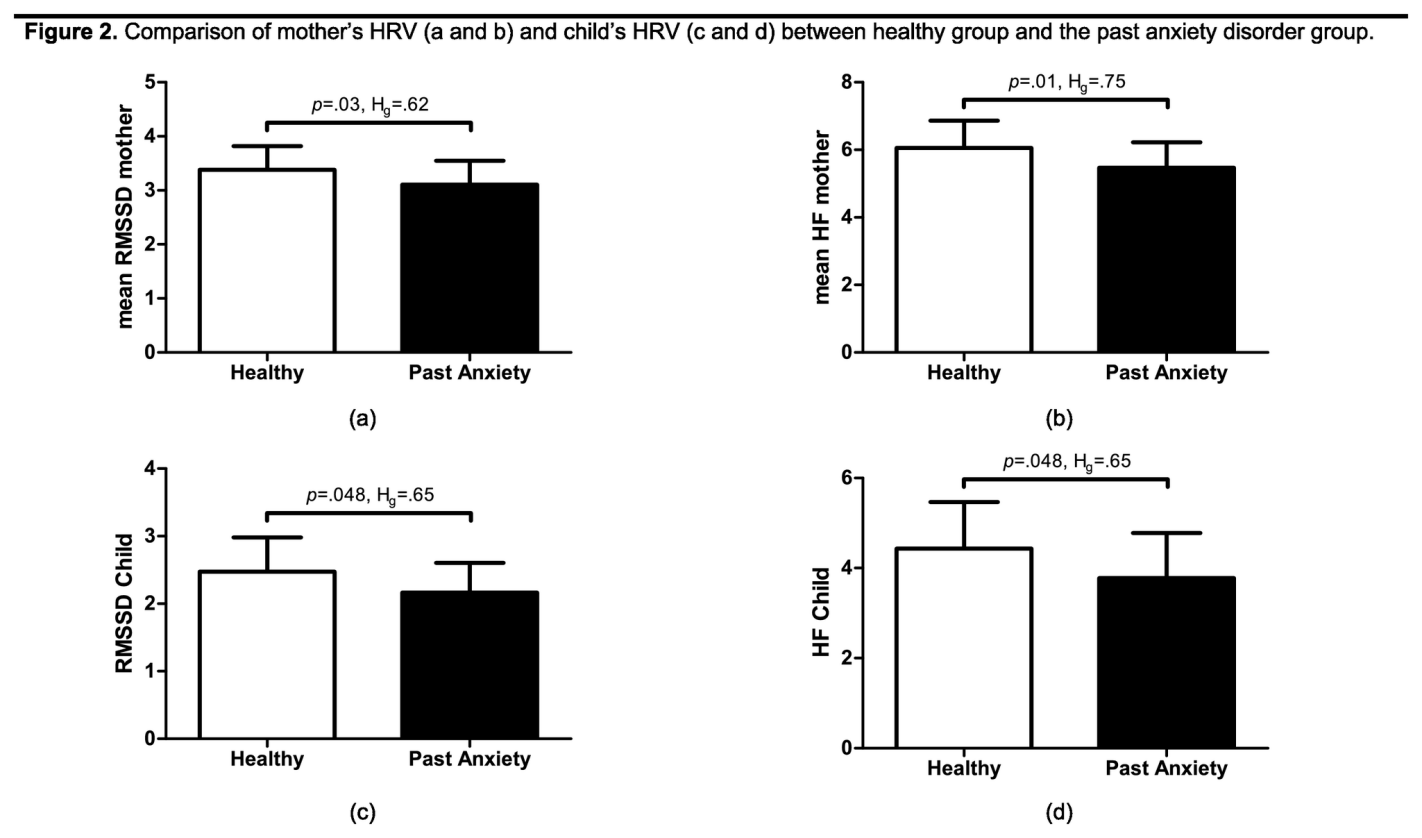
Comparison of between healthy group and the past anxiety disorder group. This figure shows for both mother and child the differences in HRV between the healthy group and the past anxiety disorder group.

Repeated measures ANOVA analysis, adjusted for mother’s age and pre-pregnancy BMI, revealed a significant between-subjects effect of group (i.e. PAD or healthy) on both RMSSD HRV (RMSSD: *F*(1,52)=4.37, *p*=.04, partial η^2^=0.08) and HF HRV (HF: F(1,52)=6.68, *p*=.01, partial η^2^=0.12), indicating reduced HRV in women with PAD (Figure 2, a and b). In addition, there was a significant within-subjects effect of phase, indicating that mothers’ HRV was significantly different across phases (RMSSD: *F*(1,52)=11.16, *p*<.001, partial η^2^=0.18 HF: F(1,52)=5.73, *p*<.001, partial η^2^=0.10) (see Table 1). There was no interaction effect of group by phase, therefore, subsequent analyses are based on the mean HRV over all five phases, since the aim of our study was to study differences between women with a PAD and the healthy group. Between-subjects effects of group remained significant for HF HRV (*F*(1,36)=4.23, *p*=.048, partial η^2^=0.12), but not for RMSSD (*F*(1,36)=1.60, *p*=.22, partial η^2^=0.05) when tests were run on the propensity score matched sample.

### Associations Between Maternal and Offspring HRV

Regression analysis, adjusted for mother’s state anxiety, offspring’s sex and age, revealed a significant group x mother’s HRV interaction effect on offspring’s HRV RMSSD (β=0.98, *p*=.01) and HRV HF (β=0.95, *p*=.046), indicating that there is a positive significant association between maternal and offspring HRV in the PAD group, but not in the healthy group. Adding birth weight and gestation length as additional covariates showed that the relationship between maternal and infant HRV was not influenced by changes in birth weight and gestational age.

### Impact of Maternal Past Anxiety Disorder on Offspring HRV

An independent *t*-test showed that children of mothers with a PAD have lower HRV than those born to mothers without a PAD (RMSSD *t*(42)=2.04, *p*=.048, H_g_=.65; HF *t*(42)=2.05, *p*=.048, H_g_=.65; Table 1 and Figure 2, c and d). Maternal state anxiety was inversely correlated with offspring RMSSD HRV but this had marginal significance (*r*=-0.27, *p*=.08), while the correlation for HF HRV was significant (*r*=-0.33, *p*=.03). These were correlations in the total group (consisting of the healthy group and PAD group); no correlations were found between maternal state anxiety and offspring HRV when focusing on groups separately (*p*>.05).

### Relationship between Offspring Temperament and Offspring HRV

Logistic regression analyses controlling for age, sex and gestation length indicated that the children’s HRV at 2-4 months of age was inversely associated with measures of their fearfulness carried out at 9-10 months of age (RMSSD: odds ratio=0.01, *p*=.047; HF: odds ratio=0.20, *p*=.07). Bootstrapped regression analyses with fearfulness as a continuous variable also indicated a significant effect of offspring HRV on fearfulness (RMSSD: 95% CI=[-1.63, -1.19]; HF: 95% CI=[-0.09, -0.01]).

## Discussion

To our knowledge, this is the first study to show that pregnant women with a PAD have autonomic abnormalities early in pregnancy and that these abnormalities may impact on the future physiological (reduced parasympathetic function) and mental (fearfulness) wellbeing of their offspring. Pregnant women with PAD displayed reduced parasympathetic nervous system activity (indexed by HRV), compared to those without PAD. Furthermore, the offspring of mothers with a PAD also had lower HRV than those whose mothers had been healthy. In the group of mothers with PAD, an association between maternal HRV and offspring HRV was observed. In the offspring, lower HRV at 2-4 months was associated with a fearful temperament assessed approximately 7 months later. These findings were independent of variations in state-anxiety, age, sex, BMI and mother-child associations were not explained by the children’s birth weight or gestational age. 

Consistent with prior research, this study shows that people with past psychiatric disorders have lower HRV [6-8,31]. The novel contribution we make here is the result that pregnant women with a past anxiety disorder also have reduced HRV and these psychophysiological alterations may impact on the future physiological and mental wellbeing of their offspring. It is important to note that the HF HRV differences between women with and without PAD – findings associated with a moderate to large effect size – remained after controlling for confounding factors including state anxiety, age and BMI by propensity score matching. Future studies are needed to confirm this finding in a larger sample in order to generalize to both pregnant and non-pregnant women populations. We note here that women with PAD have a higher level of a subclinical trait (e.g., anxiety), although it no longer reaches criteria for clinical diagnosis. Although we controlled for state anxiety as a confounding factor, it is possible that state anxiety also impacts on maternal HRV as well as the physiological and mental state of the offspring.

There are numerous possible means by which maternal PAD and/or HRV might be associated with parasympathetic nervous system function in the offspring. Clearly, mothers and their children share genes and environmental exposures and the health of mothers in pregnancy is an important determinant of child development and health. Maternal behavior may also be an important factor in this association [32-34]. In our study, the mothers with a PAD not only had significantly lower HRV compared to the healthy group, but also had altered state anxiety, albeit to a lesser extent than in the past. In comparison to the healthy group, the offspring of mothers with a PAD also had lower HRV. Lower HRV in all children was shown to be associated with a fearful temperament. Therefore, it is possible that mothers with a PAD and their offspring share an underlying psychological disorder, which might explain links between maternal and offspring HRV. Our study highlights the need for more research into how mothers might transmit their pro-anxiety phenotype to their offspring.

A variety of genes influence vulnerability to anxiety disorders. These include serotonin-transporter linked polymorphic region (*5-HTTLPR*), Catechol-O-methyltransferase (*COMT*), and brain-derived neurotrophic factor (BDNF) gene variants [35]. We have reported previously that the combination of a BDNF V/V genotype and early life stress predicts changes in brain structure that are associated with lower HRV and higher anxiety [36]. These findings provide a potential explanation for our observed relationship between HRV of mothers with a PAD and their children, but not in healthy controls.

Another possibility is that altered autonomic function in pregnant mothers may impact on the development of their offspring, as a form of developmental programming. Reduced HRV is associated with dysregulation of various allostatic systems, including glucose regulation, hypothalamic-pituitary-adrenal (HPA) axis function and inflammatory processes [37], all of which may program fetal development [38-44]. However, it is not clear whether the abnormal ANS function, reflected by lower HRV, is an important causative factor in these disorders, or simply the result of shared underlying processes. 

Observed fearfulness in the children at 9 months was associated with their HRV at 2-4 months. It is possible that 9 month olds with higher fearfulness already had characteristics of a fearful temperament at 2-4 months, which could explain the association between reduced HRV and future fearful temperament as a persistence of these related factors. However, it is very challenging, if not impossible, to detect these psychological features in young infants [45,46]. Therefore, we were unable to determine whether reduced HRV came before the development of fearfulness or not, making it difficult to infer causation in this association. Nevertheless, reduced HRV in infancy may be an early risk marker for the development of psychological abnormalities, such as fearfulness, in later life. This observation makes an important contribution to the literature, which indicates strong associations between low HRV and fearful temperament (reflected in inhibited behavior, shyness, harm avoidance, anxiety and distress) [45,47,48]. Such behavioral characteristics have been linked to internalizing problems and psychiatric diagnoses [49,50]. Low HRV may indicate a vulnerability to the development of psychopathology, as suggested by our observation that infant’s HRV is related to later fearfulness.

Our study has a number of advantages, including a prospective design and longitudinal assessment; assessment of maternal HRV during the first 14 weeks of pregnancy, which is the period of greatest fetal developmental vulnerability to external influences; exclusion of women on antidepressants, which is an important consideration in studies of HRV; and use of an infant temperament (fearfulness) measure, which avoids bias in parental perceptions.

In summary, our study showed that pregnant women with a PAD had altered ANS function that was also found in their infants. Alterations in the offspring were associated with fearfulness in later infancy, suggesting transmission of a pro-anxiety phenotype from mothers to their children. Determination of the mechanisms of this transmission should be an important goal of future research. Our findings may have implications for identifying early risk factors in childhood for the development of psychological disorders in later life. Thus, future research might build on our findings to establish novel risk factors for psychopathology in childhood and use those factors to study the etiology of psychiatric disease in children.
